# Exercise training elicits superior metabolic effects when performed in the afternoon compared to morning in metabolically compromised humans

**DOI:** 10.14814/phy2.14669

**Published:** 2020-12-23

**Authors:** Rodrigo Mancilla, Bram Brouwers, Vera B. Schrauwen‐Hinderling, Matthijs K. C. Hesselink, Joris Hoeks, Patrick Schrauwen

**Affiliations:** ^1^ NUTRIM School of Nutrition and Translational Research in Metabolism Maastricht University Medical Center Maastricht The Netherlands; ^2^ Department of Nutrition and Movement Sciences Maastricht University Medical Center Maastricht The Netherlands; ^3^ Department of Radiology Maastricht University Medical Center Maastricht The Netherlands

**Keywords:** adipose tissue insulin sensitivity, hyperinsulinemic‐euglycemic clamp, skeletal muscle insulin sensitivity, timing of exercise

## Abstract

The circadian clock and metabolism are tightly intertwined. Hence, the specific timing of interventions that target metabolic changes may affect their efficacy. Here we retrospectively compared the metabolic health effects of morning versus afternoon exercise training in metabolically compromised subjects enrolled in a 12‐week exercise training program. Thirty‐two adult males (58 ± 7 yrs) at risk for or diagnosed with type 2 diabetes performed 12 weeks of supervised exercise training either in the morning (8.00–10.00 a.m., *N* = 12) or in the afternoon (3.00–6.00 p.m., *N* = 20). Compared to participants who trained in the morning, participants who trained in the afternoon experienced superior beneficial effects of exercise training on peripheral insulin sensitivity (+5.2 ± 6.4 vs. −0.5 ± 5.4 μmol/min/kgFFM, *p* = .03), insulin‐mediated suppression of adipose tissue lipolysis (−4.5 ± 13.7% vs. +5.9 ± 11%, *p* = .04), fasting plasma glucose levels (−0.3 ± 1.0 vs. +0.5 ± 0.8 mmol/l, *p* = .02), exercise performance (+0.40 ± 0.2 vs. +0.2 ± 0.1 W/kg, *p* = .05) and fat mass (−1.2 ± 1.3 vs. −0.2 ± 1.0 kg, *p* = .03). In addition, exercise training in the afternoon also tended to elicit superior effects on basal hepatic glucose output (*p* = .057). Our findings suggest that metabolically compromised subjects may reap more pronounced metabolic benefits from exercise training when this training is performed in the afternoon versus morning.

**ClinicalTrials.gov ID:**

NCT01317576.

## Graphical abstract

Afternoon exercise is more optimal than morning to improve insulin sensitivity, body composition, and exercise performance in metabolically compromised individuals.

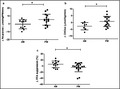

**Key points**

- The circadian clock and metabolism are tightly intertwined. Hence, the specific timing of interventions that target metabolic changes may affect their efficacy.
- As exercise training is the first‐line strategy to improve insulin sensitivity in humans, here we compared the metabolic health effects of morning versus afternoon exercise training in metabolically compromised subjects.
- Compared to participants who trained in the morning, participants who trained in the afternoon experienced superior beneficial effects of exercise training on insulin‐stimulated peripheral glucose disposal, insulin‐mediated suppression of adipose tissue lipolysis, fasting plasma glucose levels, exercise performance, and fat mass. In addition, exercise training in the afternoon also tended to elicit superior effects on basal hepatic glucose output.
- Our findings suggest that the timing of an exercise training session is a crucial environmental cue when aiming to improve glucose homeostasis in metabolically compromised subjects and elucidates that performing afternoon exercise training might be more optimal than exercising at morning hours

## 1 INTRODUCTION

To cope with the dark and light cycles on earth and the alternating periods of feeding and fasting, and activity and recovery, the human body contains a biological clock that triggers anticipatory responses by setting circadian rhythmicity. This circadian rhythmicity is regulated by a central clock, which is located in the hypothalamic suprachiasmatic nucleus (SCN), but also by multiple peripheral molecular clocks, located in distal organs such as skeletal muscle, liver, and adipose tissue (Dibner et al., 2010). While the central clock is entrained by daily light–dark cycles, the peripheral molecular clocks are robustly entrained by non‐photic environmental signals (zeitgebers) such as exercise and feeding (Chaix et al., 2016). Consequently, the central and peripheral clocks can desynchronize when cues derived from these behavioral zeitgebers are not aligned to the environmental light/dark cycle, eventually contributing to the development of insulin resistance (Morris et al., 2015; West et al., 2017).

In that context, skeletal muscle insulin sensitivity is characterized by diurnal variations in healthy humans (Van Cauter et al., 1991; Kalsbeek et al., 2014), exhibiting higher insulin‐stimulated plasma glucose clearance in the morning compared to the evening (Verrillo et al., 1989). Interestingly, we (Wefers et al., 2018) and others (Rao et al., 2015; Scheer et al., 2009) have shown that a rapid day–night shift, as a model for shiftwork or jetlag, leads to disturbed glucose homeostasis and skeletal muscle insulin resistance in humans, illustrating the indisputable role that central and peripheral molecular clocks play in regulating systemic energy homeostasis. As such, integrating different external cues at optimal times can potentially be used to improve metabolic health.

Exercise training is the first‐line strategy to counteract skeletal muscle insulin resistance and ameliorate elevated plasma glucose levels (Blair et al., 2012). Indeed, we (Brouwers et al., 2018; Meex et al., 2010) and others (Hey‐Mogensen et al., 2010; Menshikova et al., 2017) have previously elucidated that exercise training programs of 2 months or longer result in training adaptations that improve skeletal muscle insulin resistance and mitochondrial function in obese volunteers and type 2 diabetes (T2D) patients. The recent insights into the role of the circadian clock in the etiology of T2DM have raised the suggestion that the timing of exercise may affect the training‐mediated effects on glucose homeostasis. Somewhat supportive of this notion, Savikj et al. reported that consecutive bouts of high‐intensity interval exercise during 2 weeks acutely induce more beneficial 24h glycaemic profiles in T2DM subjects when performed in the afternoon as compared to a morning training regime (Savikj et al., 2019). However, so far it has not been revealed in humans if the classical, more sustained metabolic health effects of prolonged exercise training, including beneficial training‐adaptive effects on insulin sensitivity, exercise performance, and body composition, are influenced by the timing of the exercise training sessions.

To test the hypothesis that the timing of exercise affects long‐term metabolic health training adaptations in metabolically compromised individuals, we retrospectively analyzed data from a study investigating the effect of exercise training on a large range of metabolic health outcomes (Brouwers et al., 2018). In this previous study, volunteers performed regular, supervised exercise training for 12 weeks either in the morning or afternoon but the effect of the timing of exercise was not previously analyzed (Brouwers et al., 2018). Here, we report that exercise performed in the afternoon leads to greater improvements in skeletal muscle and adipose tissue insulin sensitivity in conjunction with other beneficial metabolic health effects such as larger declines in fat mass and greater improvements in exercise performance when compared to morning training.

## 2 MATERIALS AND METHODS

### 2.1 Ethical approval

All subjects gave their written informed consent. The protocol was reviewed and approved by the institutional medical ethics committee and the study was carried out at Maastricht University Medical Center, The Netherlands, following the declaration of Helsinki principles (ClinicalTrials.gov ID: NCT01317576).

### 2.2 Subjects

Thirty‐five males with a BMI > 26 kg/m^2^ participated in a 12‐week exercise training program. The baseline, metabolic characteristics of participants were previously reported in (Brouwers et al., 2017), whereas the effects of exercise training in obese and NAFL volunteers were reported in (Brouwers et al., 2018). The study was not designed to investigate the effect of the timing of exercise and the reason for subjects performing exercise in the morning or evening was depending on the scheduling possibilities and personal preferences. For the current analysis, 32 volunteers were included that performed ALL of their exercise training sessions in either the morning (AM, *N* = 12) or the afternoon (PM, *N* = 20). Overall, 11 subjects were classified as overweight or obese, 9 subjects had a non‐alcoholic fatty liver (defined as liver fat ≥5% measured by proton magnetic resonance spectroscopy [^1^H‐MRS], and 12 subjects were classified as well‐controlled type 2 diabetics patients on oral medication; those groups were equally distributed over the AM and PM groups according to scheduling possibilities and personal preference where possible.

Unstable body weight (body weight variance of >3 kg in the last 3 months before enrolling the study), impaired renal and cardiac function, use of anti‐thrombotic medication and beta‐blockers, elevated resting blood pressure (>160/100 mmHg), and previously undergoing insulin therapy, were exclusion criteria for this study. Detailed inclusion criteria were reported previously by Brouwers et al. (Brouwers et al., 2018). The medication use did not change during the study, but antidiabetic medication was stopped 7 days before the hyperinsulinemic‐euglycemic clamp.

Before, during, and after 12 weeks of exercise training maximal workload (Wmax) and maximal aerobic capacity (VO_2_max) were determined during a graded cycling test with ECG monitoring until exhaustion (Kuipers et al., 1985). Body composition was assessed by dual X‐ray absorptiometry (Hologic Discovery A, Waltham, MA, USA). Participants were asked to maintain their regular dietary behavior throughout the study and to consume the same evening meal prior to the hyperinsulinemic‐euglycemic clamp test before and after training.

### 2.3 Exercise training protocol

Subjects enrolled in a tightly controlled progressive exercise program for 12 weeks, combining aerobic‐type and resistance‐type exercises with three sessions per week. Twelve subjects performed all their exercise training sessions between 08:00 and 10:00 a.m. (AM group), whereas 20 subjects performed all training sessions between 15:00 and 18:00 PM (PM group). Compliance with the exercise training averaged 98%. All subjects performed twice a week aerobic exercise on a cycling ergometer for 30 min at 70% of a previously determined workload (Wmax), and once a week resistance exercise comprised of three series of 10 repetitions at 60% of the subject's previously determined maximal voluntary contraction (MVC). Resistance‐type exercises targeted large muscle groups from the low and high limbs (leg extension, leg press, chess press, lat pull down, triceps and biceps curls, abdominal crunches, and horizontal row). MVC was derived from five multiple repeated maximum testing. Total muscle strength was calculated as the sum of the derived MVC for all eight muscle groups. Each training session involved a warming‐up and cooling down phase by cycling 5 min on the ergometer at 45% Wmax at the beginning and at the end of the training session, respectively. Both muscle strength and Wmax were reassessed every 4 weeks to readjust training loads according to adopted exercise capacities. Training sessions were fully supervised and performed with 3–4 individuals at a time.

### 2.4 Hyperinsulinemic‐euglycemic clamp

A 2‐step hyperinsulinemic‐euglycemic clamp (10 mU/m^2^/min and 40 mU kg^‐1^ min^‐1^) was performed before and after the training period as originally described (DeFronzo et al., 1979). Dietary habits were stable and physical activity was avoided during the 2 days prior to the clamp, which was performed 4 days before starting the regular training period and 48–72 hr after the last training session to prevent the carry‐over effect of the last exercise bout. Subjects were assisted to the laboratory after an overnight fast and clamp started with a primed continuous infusion of [6,6‐2H2]‐glucose (0.04 mg kg^‐1^ min^‐1^) to determine the rates of endogenous glucose production (EGP), glucose appearance (Ra), and glucose disposal (Rd) as described previously (Brouwers et al., 2017, 2018). After 3 hr of continuous glucose tracer infusion, a primed constant infusion of insulin (10 mU/m2/min) started for 4 hr, followed by high dose of insulin (40 mU/m2/min) infused continuously for 2 hr. During the last 30 min of the non‐insulin stimulated period (t = 150–180) and under both insulin‐stimulated steady states (t = 390–420 and t = 510–540), blood samples were collected and substrate utilization was measured by indirect calorimetry (Omnical, Maastricht Instruments, The Netherlands).

### 2.5 Calculations

Steele's single‐pool non‐steady‐state equations were used to calculate glucose rate of appearance (Ra) and glucose rate of disposal (Rd) (Steele, 1959). The distribution volume of glucose was assumed to be 0.160 L/kg. Insulin‐stimulated glucose disposal was computed as the difference between Rd under insulin‐stimulated conditions (40 mU/m^2^/min) and under basal non‐insulin‐stimulated conditions (delta Rd). Glucose oxidation was computed with the assumption that protein oxidation was negligible, using Peronnet's equation (Peronnet & Massicotte, 1991). Non‐oxidative glucose disposal (NOGD) was calculated as Rd (40 mU/m^2^/min) – glucose oxidation (40 mU/m^2^/min). Liver insulin sensitivity was computed as percent suppression of EGP during low dose (10 mU/m^2^/min) insulin infusion. Adipose tissue insulin sensitivity was computed as percent suppression of plasma‐free fatty acids during low dose (10 mU/m^2^/min) insulin infusion.

### 2.6 Blood sample analysis

Isotopic enrichment of plasma glucose was determined by electron ionization gas chromatography‐mass spectroscopy (Ackermans et al., 2001). Arterialized blood samples were collected and immediately centrifuged at high speed. Plasma was frozen in liquid nitrogen and stored at −80°C until assayed. Plasma glucose and free fatty acids levels were determined with enzymatic assays automated on a Cobas Fata/Mira (FFA: Wako NEFA C test kit; Wako Chemicals, Neuss, Germany) (Plasma Glucose: Hexokinase method; La Roche, Basel, Switzerland).

### 2.7 Statistics

Data are presented as mean ± standard deviation (*SD*). Shapiro–Wilk normality test was carried out to evaluate the normal distribution. An unpaired Student's *t* test was used to compare baseline subjects’ characteristics for the morning and afternoon exerciser group. Post minus pre‐exercise training changes in primary (whole‐body insulin sensitivity) and secondary outcome parameters (exercise performance and body composition) were compared between the morning and afternoon exercise groups using unpaired Student's *t* test. A *p* value ≤ .05 was considered statistically significant. Statistical analyses were performed using SPSS 25.0 for Windows.

## 3 RESULTS

### 3.1 Baseline characteristics

Table 1 shows the baseline subject characteristics of the volunteers that were included in the current analysis, classified by the timing of exercise training either in the morning (AM) or afternoon training (PM). No significant differences in age, body weight, BMI, and body composition were observed at baseline between the AM and PM groups (all *p* > .05, Table 1). In addition, individuals with overweight or obesity, individuals with NAFL, and individuals with T2DM were equally distributed over the two groups. Furthermore, baseline maximal aerobic capacity (VO_2_max), and maximal power output (Wmax) were comparable between AM and PM groups (table 1). Also, fasting plasma glucose and free fatty acids levels were comparable between AM and PM groups at baseline (*p* > .05, Table 1). No differences in any measures of insulin sensitivity were observed between AM and PM groups before exercise training (all *p* > .05, Table 1).

**Table 1:** Baseline subjects characteristics

	AM	PM
Sample size	12	20
T2D subjects	4	8
NAFL subjects	3	6
Healthy obese subjects	5	6
Age (year)	61 ± 5	57 ± 7
Body weight (kg)	94.7 ± 11.7	98.1 ± 10
BMI (kg/m^2^)	30.3 ± 2.6	29.8 ± 2.3
Fat mass (kg)	27.4 ± 4.3	28.8 ± 5.6
Fat percentage (%)	28.6 ± 2.3	29 ± 3.2
Trunk fat mass (kg)	16.0 ± 2.5	16.2 ± 3.4
Fat‐free mass (kg)	65.4 ± 7.2	67.1 ± 5.1
VO2_max_ (ml/kg/min)	26 ± 4.0	26.5 ± 4.5
W_max_ (W/kg)	1.9 ± 0.4	2.0 ± 0.3
Fasting glucose (mmol/l)	6.7 ± 2.1	6.8 ± 2.1
Fasting‐free fatty acids (µmol/l)	566 ± 171	615 ± 169
Clamp data		
Basal EGP (µmol/min/kgFFM)	7.4 ± 1.5	8.1 ± 3.5
Basal R_d_ (µmol/min/kgFFM)	7.8 ± 1.8	7.9 ± 3.6
Basal CHO_ox_ (µmol/min/kgFFM)	5.9 ± 3.9	5.3 ± 2.6
Insulin‐induced suppression of plasma FFA (%)	−62.6 ± 9.5	−61.6 ± 16.0
Insulin‐induced suppression of EGP (%)	−41.8 ± 26.3	−32.2 ± 28.9
Delta R_d_ (µmol/min/kgFFM)	16.9 ± 11.2	15.2 ± 9.0
Delta NOGD (μmol/min/kgFFM)	8.9 ± 7.2	8.2 ± 6.2

Note

Data are displayed as mean ± *SD*. BMI, body mass index; VO2_max_, maximal aerobic capacity; W_max_, maximal power output; EGP, endogenous glucose production; R_d_, glucose disposal; CHO_ox_, carbohydrate oxidation; FAT_ox_, fat oxidation; FFA, free fatty acids; NOGD, non‐oxidative glucose disposal. Insulin‐induced suppression of EGP and plasma FFA was measured at 10 mU/m^2^/min. Delta Rd was calculated as the difference between basal and insulin‐stimulated glucose disposal at 40 mU/m^2^/min infusion rate.

### 3.2 Effect of timing on the metabolic consequences of exercise‐training

#### 3.2.1 Afternoon exercise training improves peripheral insulin sensitivity and glucose homeostasis more profoundly than morning exercise training

**Figure 1:**
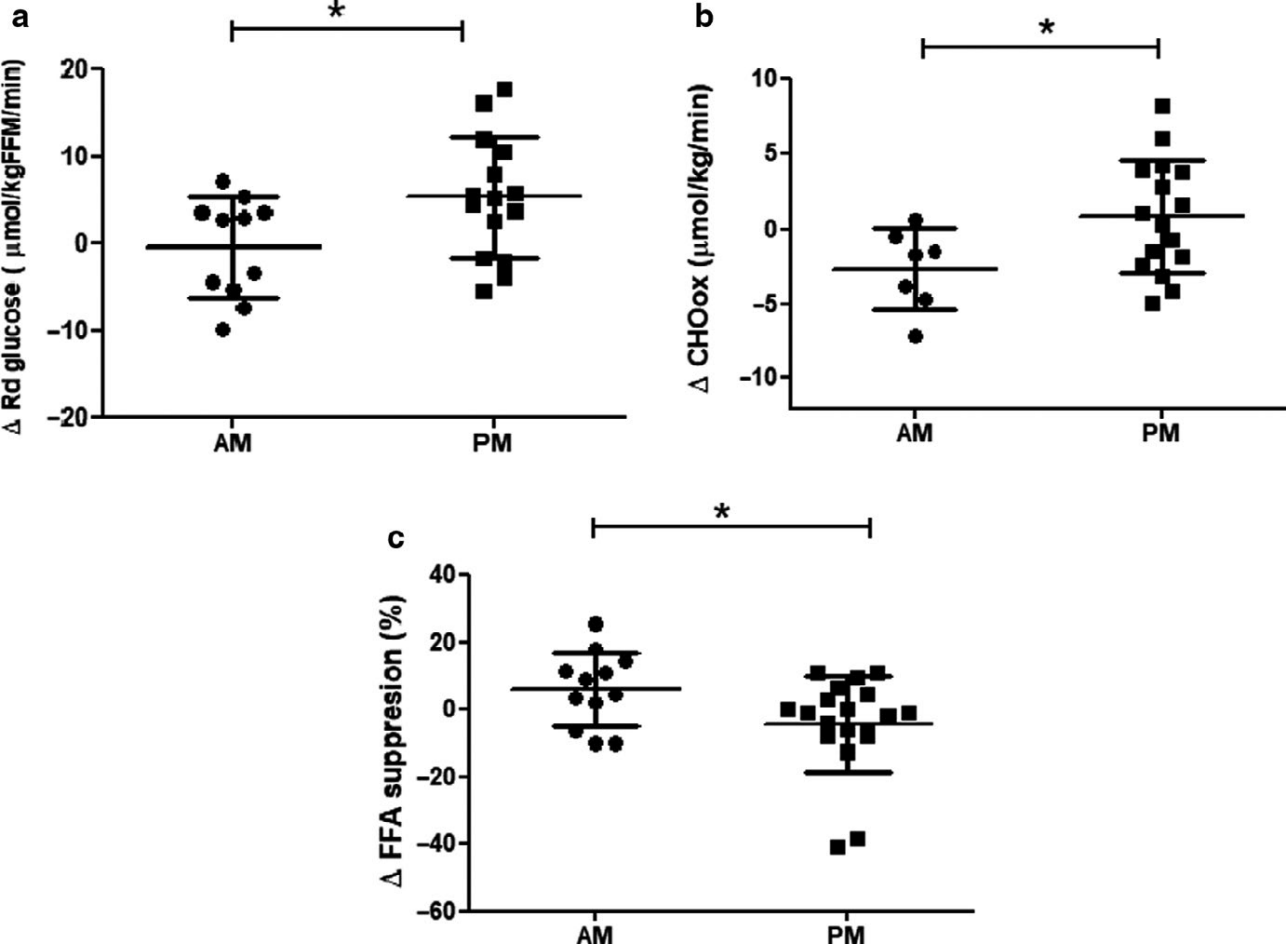
Changes in peripheral insulin sensitivity and glucose oxidation upon the timing of exercise training either in the morning (AM) or in the afternoon (PM). (a) Changes in peripheral insulin‐stimulated glucose disposal (from basal to insulin‐stimulated) were significantly greater in the PM group compared to the AM group. Data are expressed as means ± *SD*. *p < .05. *n* = 11 for the AM group and *n* = 15 for the PM group. (b) Changes in insulin‐stimulated glucose oxidation (from basal to insulin‐stimulated) were significantly greater in the PM group compared to the AM group. Data are expressed as means ± *SD*. *p < .05. *n* = 7 for the AM group and *n* = 16 for the PM group. (c) Changes in insulin‐mediated suppression of plasma‐free fatty acids (%) improved significantly more in the PM group compared to the AM group. Data are expressed as means ± *SD*. *p < .05. *n* = 12 for the AM group and *n* = 19 for the PM group

Training‐induced effects on insulin‐stimulated peripheral glucose uptake, as expressed by the changes in plasma glucose disposal (delta R_d_) from basal to insulin‐stimulated conditions, were significantly affected by the timing of exercise (*p* = .03, Figure 1a, Table 2). Specifically, training‐induced effects on delta R_d_ were greater in the PM group compared to the AM group (+5.2 ± 6.4 vs. −0.5 ± 5.5 μmol/min/kgFFM, in PM vs. AM). The superior effect of PM exercise training on insulin‐stimulated glucose disposal was also reflected in a larger increase in training‐induced change in glucose oxidation upon insulin effects (+0.8 ± 3.3 μmol/min/kgFFM in PM vs. −2.6 ± 2.4 μmol/min/kgFFM in AM, *p* = .04, respectively) (Figure 1b, Table 2), whereas insulin‐stimulated non‐oxidative glucose disposal (NOGD) was not significantly affected by the timing of exercise (*p* = .1, Table 2). It should be noted though, that due to technical issues with tracer handling, the data on Rd (*n* = 15 in PM) as well as glucose oxidation and NOGD (*n* = 7 in AM and *n* = 16 in AM and PM) are based on smaller group sizes.

**Table 2:** Effects of morning and afternoon exercise training on insulin sensitivity and metabolic health

	AM		AM Δ	PM		PM Δ	*p* value
	Pre	Post		Pre	Post		
BMI (kg/m^2^)	30.3 ± 2.6	30.5 ± 2.5	0.2 ± 0.5	29.8 ± 2.3	29.6 ± 2.4	−0.2 ± 0.8	0.11
Body weight (kg)	94.7 ± 11.7	95.3 ± 11.6	0.7 ± 1.6	98.1 ± 10.0	97.5 ± 10.5	−0.6 ± 2.5	0.11
Fat mass (kg)	27.4 ± 4.3	27.2 ± 4.7	−0.2 ± 1.0	28.8 ± 5.6	27.6 ± 5.7	−1.2 ± 1.3	**0.03**
Fat‐free mass (kg)	65.4 ± 7.2	65.8 ± 7.7	0.4 ± 1.0	67.1 ± 5.1	67.4 ± 5.6	0.3 ± 1.5	0.82
Fat percentage (%)	28.6 ± 2.3	28.3 ± 2.4	−0.3 ± 0.7	29 ± 3.2	28 ± 3.1	−1.0 ± 0.9	**0.03**
Trunk fat mass (kg)	16.0 ± 2.5	16 ± 2.8	−0.07 ± 0.7	16.2 ± 3.4	15.4 ± 3.3	−0.7 ± 1.0	0.059
VO2max (ml/kg/min)	26.0 ± 4.0	27.7 ± 3.0	1.8 ± 2.2	26.5 ± 4.5	29.5 ± 5.0	3.0 ± 2.1	0.18
Wmax (W/kg)	1.9 ± 0.4	2.1 ± 0.3	0.2 ± 0.1	2.0 ± 0.3	2.4 ± 0.4	0.4 ± 0.2	**0.05**
Total muscle strength (kg)	79.4 ± 14	91.7 ± 12.3	12.3 ± 7.5	82.5 ± 15.8	98.4 ± 20.7	15.9 ± 10.3	0.30
Fasting‐free fatty acids (µmol/l)	567 ± 171	547 ± 174	−19.8 ± 139	615 ± 170	507 ± 177	−122 ± 198	0.13
Fasting glucose (mmol/l)	6.7 ± 2.1	7.2 ± 2.7	0.5 ± 0.8	6.8 ± 2.1	6.4 ± 2.0	−0.3 ± 1.0	**0.02**
Clamp data							
Basal EGP (µmol/min/kgFFM)	7.4 ± 1.5	8.5 ± 1.2	1.0 ± 1.6	8.1 ± 3.5	8.1 ± 3.7	−0.01 ± 0.9	0.057
Basal Rd (μmol/min/kgFFM)	7.8 ± 1.8	9.4 ± 2.5	1.0 ± 1.5	7.9 ± 3.6	7.7 ± 4.2	−0.3 ± 2.4	0.17
Basal CHOox (μmol/min/kgFFM)	5.9 ± 3.9	5.5 ± 3.2	0.2 ± 3.0	5.3 ± 2.6	5.6 ± 2.3	0.1 ± 2.1	0.89
Insulin‐induced suppression EGP (%)	−41.8 ± 26.3	−42.8 ± 31.0	−2.3 ± 28.6	−32.2 ± 28.9	−40.8 ± 29.0	−10.6 ± 25.1	0.44
Insulin‐induced suppression plasma FFA (%)	−62.6 ± 9.5	−56.7 ± 15.2	5.9 ± 11.0	−61.6 ± 16.0	−65.8 ± 12.4	−4.5 ± 13.7	**0.04**
Delta Rd (μmol/min/kgFFM)	16.9 ± 11.2	14.5 ± 13.8	−0.5 ± 5.5	15.2 ± 9.0	20.2 ± 13.6	5.2 ± 6.4	**0.03**
Delta CHOox (μmol/min/kgFFM)	6.7 ± 4.0	5.5 ± 3.8	−2.6 ± 2.4	6.3 ± 2.8	7.4 ± 4.0	0.8 ± 3.3	**0.04**
Delta NOGD (μmol/min/kgFFM)	8.9 ± 7.2	8.5 ± 12.0	−0.9 ± 5.3	8.2 ± 6.2	13.4 ± 10.5	4.2 ± 5.4	0.16
Delta FATox (μmol/min/kgFFM)	−1.8 ± 1.1	−1.6 ± 1.0	0.4 ± 0.5	−1.8 ± 0.7	−1.9 ± 1.0	−0.04 ± 0.7	0.24

Note

Data are displayed as mean ± *SD*. BMI, body mass index; VO2_max_, maximal aerobic capacity; W_max_, maximal power output; EGP, endogenous glucose production; R_d_, glucose disposal; CHO_ox_, carbohydrate oxidation; FAT_ox_, fat oxidation; FFA, free fatty acids; NOGD, non‐oxidative glucose disposal. Insulin‐induced suppression of EGP and plasma FFA was measured at 10 mU/m^2^/min infusion rate. Delta Rd was calculated as the difference between basal and insulin‐stimulated glucose disposal at 40 mU/m^2^/min infusion rate. Bold font is used to indicate statistically significant differences between groups.

The effect of exercise training on insulin‐mediated suppression of plasma FFA (%FFA suppression), a marker of adipose tissue insulin sensitivity, was significantly larger in the PM versus AM group (−4.5 ± 13.7% vs. +5.9 ± 11% in PM vs. AM, *p* = .04, Figure 1c, Table 2), indicating that the effect of PM training on adipose tissue insulin sensitivity was more pronounced than AM training. However, changes in fasting plasma FFA levels upon exercise training did not reach statistical significance upon comparing PM and AM (−122.3 ± 198.6 µmol/l vs. −19.8 ± 139.8 in PM vs. AM, *p* = .13, Table 2).

Training‐induced changes in basal endogenous glucose production tended to be different between afternoon and morning exercisers (EGP: −0.01 ± 0.9 vs. +1.08 ± 1.6 µmol/min/kgFFM in PM vs. AM, *p* = .057, Table 2) but the timing of exercise did not have significant effects on training‐induced changes in insulin‐mediated suppression of endogenous glucose production (%EGP suppression), a marker of liver insulin sensitivity (−10.6 ± 25.1 vs. −2.3 ± 28.6% in PM vs. AM, *p* = .4, Table 2).

Changes in fasting plasma glucose levels upon training were significantly affected by the timing of exercise (*p* = .02, Table 2), with a greater decline of fasting glucose levels in the PM group (−0.3 ± 1.0 mmol/l) versus AM group (+0.5 ± 0.8 mmol/l, Table 2).

#### 3.2.2 Exercise training performed in the afternoon has more profound effects on body composition than exercise training in the morning

**Figure 2:**
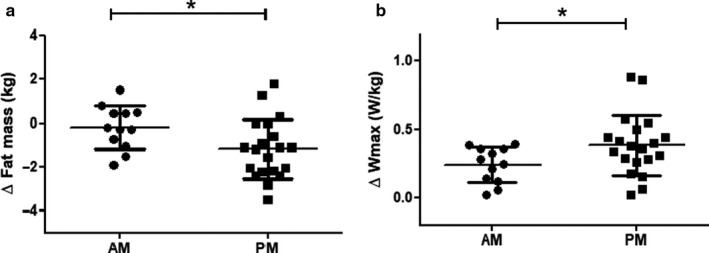
Fat mass (kg) and Wmax (W/kg) changes upon the timing of exercise training either in the morning (AM) or in the afternoon (PM). (a) Changes in Fat mass (kg) were significantly greater in the PM group compared to the AM group. Data are expressed as means ± *SD*. *p = .03. *n* = 12 for the AM group and *n* = 20 for the PM group. (b) Changes in Wmax (W/kg) were significantly greater in the PM group compared to the AM group. Data are expressed as means ± *SD*. *p = .05. *n* = 12 for the AM group and *n* = 20 for the PM group

Training‐induced reductions in fat mass (−1.2 ± 1.3 vs. −0.2 ± 1.0 kg in PM vs. AM, *p* = .03, Figure 2a, Table 2) and fat percentage (−1.0 ± 0.9 vs. −0.3 ± 0.7% in PM vs. AM, *p* = .03, Table 2) were significantly larger in the PM group compared to the AM group. Trunk fat mass tended to decrease more in the PM group compared to the AM group, although this effect did just not reach statistical significance (−0.7 ± 1.0 vs. −0.07 ± 0.7 kg in PM vs. AM, *p* = .059, Table 2).

#### 3.2.3 Exercise training performed in the afternoon induces greater improvements on exercise performance compared to exercise training performed in the morning

Maximal power output (Wmax) tended to increase more in the PM versus AM group (+36.6 ± 22 vs. +24.4 ± 11.3 Watt in PM vs. AM, *p* = .08) and this effect was statistically significant upon correction for body weight (0.40 ± 0.2 vs. 0.24 ± 0.1 W/kg in PM vs. AM, *p* = .05, Figure 2b, Table 2). VO_2_max tended to increase more in the PM group compared to the AM group, although this effect did not reach statistical significance (+3.0 ± 2.1 vs. +1.8 ± 2.2 ml kg^‐1^ min^‐1^ in PM vs. AM, *p* = .1, Table 2). Timing of exercise had no statistically significant effects on the training‐induced changes in total muscle strength, body weight, BMI, and fat‐free mass (FFM) (Table 2).

## 4 DISCUSSION

Exercise training is known to induce multiple beneficial effects on whole‐body glucose homeostasis and metabolic health, although effects are not equal in magnitude for all individuals. Exercise volume and intensity are classically recognized as crucial determinants of the beneficial effect of exercise training. Triggered by novel insights in the role of the biological clock in metabolism, it has recently been postulated that also the time of day at which acute exercise is performed affects the metabolic adaptations to exercise, but evidence in humans is scarce. Here, we demonstrate that exercise training performed in the afternoon is more beneficial for improving peripheral insulin sensitivity (skeletal muscle and adipose tissue insulin sensitivity) in comparison with exercise training in the morning. The greater improvements in peripheral insulin‐stimulated glucose disposal observed upon exercise training in the afternoon could primarily be attributed to increased insulin‐stimulated glucose oxidation and was paralleled by greater improvements in fasting plasma glucose levels and exercise performance. In addition, a superior decline of fat mass was found upon exercise training in the afternoon compared to morning exercise training. Together, these data provide evidence in humans that the timing of exercise may amplify the beneficial health effects of exercise training.

Insulin‐stimulated skeletal muscle glucose uptake is crucial to maintain normal glucose homeostasis as approximately 80% of postprandial glucose clearance resides within skeletal muscle (Shulman et al., 1990). By animal models, recent studies revealed that insulin‐stimulated skeletal muscle glucose clearance interacts with the peripheral muscle clock and such interplay can be affected by exercise timing (Ezagouri et al., 2019; Sato et al., 2019). Nevertheless, whether there is an optimal time to exercise and to reap greater improvements of insulin sensitivity in humans was unknown so far. Interestingly, we here show that exercising in the afternoon is more beneficial for skeletal muscle insulin sensitivity in comparison with exercise training in the morning. In this regard, our findings are in line with a report showing that 2 weeks of high‐intensity interval training (HIIT) in the afternoon acutely improved 24 hr glycaemic profile in subjects with T2D, while performing HIIT in the morning possessed detrimental effects on blood glucose levels throughout the day (Savikj et al., 2019). However, it is important to note that in this cross‐over design, Savikj et al. measured the effects of 2‐weeks HIIT training acutely; to the best of our knowledge, the effect of the timing of exercise sessions on more classic longer‐term training adaptations such as tissue‐specific insulin sensitivity had not been studied so far in humans. Interestingly, the superior benefits of afternoon exercise on insulin‐stimulated skeletal muscle glucose uptake compared to morning training are observed in the absence of changes in body weight in any group, reflecting more optimal local‐tissue adaptations in response to afternoon exercise timing. Whether these effects are due to an effect of exercise training on the skeletal muscle peripheral clock, on diurnal rhythms in hormonal secretion or involve other mechanisms needs to be further investigated.

In addition to skeletal muscle insulin sensitivity, hepatic glucose output and adipose tissue insulin sensitivity also contribute to the regulation of circulatory glucose levels. In this regard, it is relevant to highlight that afternoon exercise also triggered greater metabolic adaptations in other distal organs beyond those shown in skeletal muscle. Here, we reported that under hyperinsulinemic clamp conditions plasma FFA levels were significantly more suppressed by afternoon exercise training, possibly reflecting a greater antilipolytic activity of insulin in adipose tissue. Of interest, white adipose tissue FFA release and esterification feature diurnal oscillations controlled by the adipose tissue peripheral clock which responds to environmental cues such as meal ingestion and exercise timing (Kiehn et al., 2017). Uncontrolled adipose tissue lipolysis has been strongly associated with the development of skeletal muscle insulin resistance (Sparks et al., 2009) and ectopic fat accumulation (McQuaid et al., 2011). It can be speculated that a lower insulin‐mediated suppression of FFA release upon exercising in the morning could sustain FFA supply for oxidation, thus reducing glucose oxidation and diminishing insulin sensitivity compared to the effects of exercise training performed in the afternoon. Consequently, our results suggest that the timing of exercise training affects adipose tissue FFA handling and that afternoon training is more beneficial for adipose tissue insulin sensitivity than morning training.

Interestingly, we observed that exercising in the afternoon is more beneficial for fasting plasma glucose levels compared to exercise training in the morning. This differential response in fasting plasma glucose levels between the morning and afternoon training groups is consistent with the differences observed in the basal endogenous glucose production (EGP) upon exercise timing. Previous studies have indicated that the effects of exercise‐training on basal EGP are profoundly affected by the pre‐exercise nutritional status (Kjaer et al., 1990). Thus, a robust increase in hepatic glucose release, next to a rise in plasma glucose levels, was observed in subjects diagnosed with type 2 diabetes upon intense exercise in the fasted state as a consequence of aberrant increments of plasma epinephrine and glucagon synthesis (Kjaer et al., 1990). Moreover, it has been shown that hepatic glucose output is unaffected by exercising at fed conditions in subjects with T2D (Larsen et al., 1997). In the current study, pre‐exercise meal ingestion was not controlled, and exercise training was performed 2–3 hr after either breakfast or lunch. In addition, exercise may regulate appetite and thereby timing of exercise could affect post‐exercise food intake. Clearly, future studies are needed to investigate if and how pre‐ and post‐exercise food intake may affect the results of the timing of exercise on glucose homeostasis.

Exercise training performed in the afternoon also induced a more profound increase in maximal power output than in the morning. Previous studies consistently reported that exercise performance, exercise efficiency, and muscle strength display profound circadian variations (Bessot et al., 2006; Ezagouri et al., 2019), all peaking in the afternoon. In this regard, our findings indicate that optimizing exercise timing can boost exercise‐induced increases in physical performance. This gives rise to the notion that exercising in the afternoon, when muscle is primed to meet the energy demand of contraction, might trigger greater benefits. Somewhat aligned with our findings, Küüsmaa et al. previously reported that 24 weeks of combined resistance‐type and endurance‐type exercise training (2–5 times/week) executed in the afternoon induced a larger decrease in fat mass and lead to greater improvements in exercise performance (time to exhaustion) as compared to morning training in young healthy adults (Kuusmaa et al., 2016). Interestingly, here we elucidate that greater improvements of functional capacities upon afternoon exercise are observed in conjunction with superior metabolic health adaptations than morning exercise, which support the relevance of exercise timing in a clinical setting. Consistent with our findings regarding the more pronounced improvements in exercise performance in the afternoon training group, exercising in the afternoon also induced larger decreases in fat mass compared to exercising in the morning, in absence of changes in body weight.

### 4.1 Limitations of the study

The present study addressed the clinical relevance of the timing of regular exercise training by retrospectively analyzing existing research data and was consequently not designed to elucidate underlying mechanisms that might support our findings. Furthermore, the retrospective nature of the study poses some additional limitations. Thus, subjects were not randomized over the AM and PM groups, and more subjects were included in the PM group, which may have affected the outcome of the study. Moreover, the retrospective analysis may also have the advantage that results are not biased by prior knowledge on expected effects by volunteers and investigators. Also, due to the small sample size, it was impossible to separate group‐dependent metabolic adaptations upon exercise timing in healthy obese, NAFL or T2D individuals. Additionally, our investigation only included males and cannot be generalized to the entire population, although similar training‐induced metabolic adaptations, regardless of exercise timing, have been reported in adult males and females (Astorino et al., 2011). To the best of our knowledge, gender differences in the effects of the timing of exercise on health benefits have so far not been investigated in humans, and are eagerly awaited. Whether changes in food intake and dietary habits occurred during the supervised training period cannot be concluded from the present study, and cannot be excluded as part of the underlying mechanisms. However, considering that subjects were instructed to maintain their regular dietary habits throughout the training period and no significant changes in body weight were observed, our results support the notion that exercise‐induced metabolic adaptations can be optimized by performing the exercise training in the afternoon day time. To our knowledge, it is the first report about the effects of the timing of regular exercise training affecting insulin sensitivity and metabolic health in metabolically compromised subjects and T2D patients by gold standard methods, which we consider a major strength of our study.

## 5 CONCLUSIONS

In conclusion, we here show that exercise training in the afternoon leads to more pronounced exercise‐induced metabolic adaptations compared to training in the morning, in people who are metabolically compromised or have type 2 diabetes. Peripheral insulin sensitivity (skeletal muscle, adipose tissue, and hepatic glucose output), in addition to fasting plasma glucose levels, exhibited greater improvements when exercise training was performed in the afternoon (3.00–6.00 p.m.) compared to the same exercise performed in the morning (8.00–10.00 a.m.). Furthermore, afternoon exercise triggered more profound benefits on improving exercise capacity and decreasing body fat content. Our data highlight that the timing of an exercise training session is a crucial environmental cue when aiming to improve glucose homeostasis and elucidates that performing afternoon exercise training might be more optimal than exercising at morning hours. Considering the methodological limitations of the present study, future human interventions, especially larger prospective randomized controlled trials are required to confirm the present findings.

## CONFLICT OF INTERESTS

The authors declare no competing interest.

## AUTHOR CONTRIBUTIONS

R.M, M.H, J.H, and P.S conceived and designed the study. R.M, B.B, M.H, J.H, and P.S.; collected, analyzed, and interpreted the data. R.M, M.H, J.H, and P.S wrote the manuscript. R.M, B.B, V.S, M.H, J.H, and P.S revised and approved the final version of the manuscript. All authors agreed to be accountable for all aspects of the work in ensuring that questions related to the accuracy or integrity of any part of the work are appropriately investigated and resolved. All persons designated as authors qualify for authorship, and all those who qualify for authorship are listed.

## Data Availability

The data that support the findings of this study are available from the corresponding author upon reasonable request.
